# Disentangling Rheumatoid Arthritis Patients’ Implicit and Explicit Attitudes toward Methotrexate

**DOI:** 10.3389/fphar.2016.00233

**Published:** 2016-08-10

**Authors:** Annemiek J. Linn, Lisa Vandeberg, Annemarie M. Wennekers, Marcia Vervloet, Liset van Dijk, Bart J. F. van den Bemt

**Affiliations:** ^1^Amsterdam School of Communication Research, University of AmsterdamAmsterdam, Netherlands; ^2^The Netherlands Institute for Social ResearchDen Haag, Netherlands; ^3^Netherlands Institute for Health Services ResearchUtrecht, Netherlands; ^4^Department of Pharmacy and Department of Rheumatology, Sint MaartenskliniekNijmegen, Netherlands; ^5^Department of Pharmacy, Radboud University Medical CenterNijmegen, Netherlands

**Keywords:** medication adherence, implicit attitudes toward medication, rheumatoid arthritis, disease-modifying anti rheumatic drugs (DMARDs)

## Abstract

Medication non-adherence is a major public health problem that has been termed an ‘invisible epidemic.’ Non-adherence is not only associated with negative clinical consequences but can also result in substantial healthcare costs. Up to now, effective adherence interventions are scarce and a more comprehensive model of adherence determinants is required to target the determinants for not taking the medication as prescribed. Current approaches only included explicit attitudes such as self-reported evaluations of medication as determinants, neglecting the role of associative processes that shape implicit attitudes. Implicit processes can predict daily behavior more accurately than explicit attitudes. Our aim is to assess explicit and implicit attitudes toward medication and explore the relation with beliefs, adherence and clinical (laboratory) outcomes in chronically ill patients. Fifty two Rheumatic Arthritis (RA) patients’ attitudes toward Methotrexate (MTX) were explicitly (self-reported) and implicitly (Single-Category Implicit Association Test) assessed and related to the Beliefs about Medicine Questionnaire, the Compliance Questionnaire on Rheumatology and laboratory parameters [Erythrocyte Sedimentation Rate (ESR), C-Reactive Protein (CRP)]. Results show that explicit attitudes were positive and health-related. Implicit attitudes were, however, negative and sickness-related. Half of the patients displayed explicitly positive but implicitly negative attitudes. Explicit attitudes were positively related to ESR. A positive relationship between implicit attitudes and disease duration was observed. In this study, we have obtained evidence suggesting that the measurement of implicit attitudes and associations provides different information than explicit, self-reported attitudes toward medication. Since patients’ implicit attitudes deviated from explicit attitudes, we can conclude that the relationship between implicit attitudes and medication adherence is worthwhile to be further explored. With this information we can improve our understanding of the subconscious, automatic processes underlying adherence and we can develop interventions that target these implicit attitudes.

## Introduction

Non-adherence to pharmacotherapy ranges from 13 to 93%, with an average rate of 40% (Sabaté, 2003). Despite the strong relation between appropriate use of medication and health outcomes, non-adherence remains a significant problem in chronically ill patients (Neame and Hammond, 2005; Treharne et al., 2005). Knowledge of determinants associated with medication non-adherence should help researchers and health care providers to understand why patients are non-adherent. With this knowledge, interventions to improve adherence can be developed. These determinants are commonly studied using self-reports based on suggestions from cognitive expectancy-value health behavior models such as the Health Belief Model (Becker, 1974), The Common Sense Model of Self-Regulation (Leventhal et al., 2003) and the Theory of Planned Behavior (Ajzen, 1991). These theories addressing attitude-to-behavior processes share the assumption that behavior is often driven by deliberate (conscious) processes such as explicit (self-reported) beliefs or attitudes. Potentially, one reason why these models fail to successfully explain adherence (Brandes and Mullan, 2014; Rich et al., 2015) is that behavior (such as taking medication) is also influenced by unconscious processes (e.g., implicit determinants) which cannot be tapped by self-report. This project transcends these traditional models of health behavior change by focusing on implicit determinants of medication adherence.

According to the Model of Dual Attitudes (MDA) implicit attitudes guide behavior that people do not monitor consciously whereas explicit attitudes predict behavior that is more conscious (e.g., planned) (Wilson et al., 2000). When the motivation is high, and/or someone has the attention or opportunities to deliberately think about the behavior, conscious processes such as explicit (self-reported) attitudes determine behavior (Gibson, 2008). In the case of a daily behavior, such as medication adherence, associative (unconscious) processes often take over (Bargh and Chartrand, 1999). Especially when the motivation is low, or when the behavior is habitual, one is more likely to rely on these associative (unconscious) processes (Friese et al., 2006). Nonetheless, traditional theoretical models of health behavior change do not take these unconscious processes into account.

Moreover, as discussed earlier, explicit beliefs, attitudes about medication, and motivation (e.g., intentions) are often assessed using self-reports. Self-reported attitudes and behavior are susceptible to the limits of the patient’s self-knowledge and the tendency to provide socially desirable answers (Urquhart and Vrijens, 2005; Sluijs et al., 2006). In other words, studies relying on questionnaires may have a tendency to err on the optimistic side when it comes to attitudes toward medication adherence. Because of that, the adequacy of self-reports in capturing a complete picture of patients’ medication attitudes can be questioned, suggesting the need for new innovative approaches to measure patients’ determinants (Rüsch et al., 2009).

Thus, *attitudes* are shaped by two types of evaluative processes: deliberate (conscious) and associative (unconscious) processes (Gawronski and Bodenhausen, 2006). When assessing explicit attitudes, the patient deliberately evaluates and reports how positive (s)he is about the medication (i.e., good or bad) (Gawronski and Bodenhausen, 2006). Such self-reports are restricted to the limits of awareness and susceptible to response biases. Oppositely, implicit attitudes are automatically activated and occur outside the patient’s conscious awareness and control. A reaction-time task such as the Single Implicit Association Test (Greenwald et al., 1998) is able to measure automatic (implicit) *associations* between two concepts (i.e., such as the association of taking medication with being sick) which are indicators of patients’ implicit attitudes (Rüsch et al., 2009). For example, patients might know that medication prevents their disease from getting worse and report being positive about it (positive explicit attitude), but the act of taking medication might also remind the patient that (s)he is ill (negative implicit attitude). Patients have less or no insight into these associative processes (Nisbett and Wilson, 1977; Wilson and Brekke, 1994). Emerging evidence indicates that implicit attitudes explain a unique part of behavior that cannot be explained by explicit attitudes and that implicit attitudes can predict specific kinds of daily behavior more accurately than explicit attitudes (Dovidio et al., 2002; Galdi et al., 2008; Sheeran et al., 2013).

Currently, the role of implicit determinants in researching adherence has been neglected. Therefore, an aspect of non-adherence remains unexplained (Sheeran et al., 2013). Our aim is to assess both explicit *and* implicit attitudes and associations toward medication and explore their relation to self-reported beliefs, adherence and clinical outcomes.

## Materials and Methods

### Sample

Consecutive patients (>18 years old) with Rheumatic Arthritis (RA), using oral Methotrexate (MTX) who had sufficient knowledge of the Dutch language were recruited between May and July 2014 at a specialized RA hospital. They were approached when collecting their medication refill in the outpatient pharmacy. If patients agreed with participation and met these criteria, informed consent was obtained. The study was approved by the institutional review board of the research institute (number 2014-CW-33).

### Treatment Protocol

After signing informed consent, patients performed a computerized task, consisting of the implicit measures of medication attitudes and associations. Next, they filled out a questionnaire which assessed their demographics (i.e., age, educational level), years of diagnosis, explicit attitudes and associations. Clinical outcomes, co-morbidity and co-medication were obtained from patients’ medical file. This procedure was designed to avoid contamination between tasks. To avoid primed responses, respondents received little information; they were debriefed afterward.

### Measurements

#### Patients’ Attitudes, Associations and Beliefs

To measure patients’ implicit attitudes and associations, the Single Category Implicit-Association Test (SC-IAT) was used, which is a well-established and valid measure of implicit associations (Karpinski and Steinman, 2006). This is a computer-based task, designed to assess which associations are related to a certain concept in memory. The strength of an association is inferred from the time it takes them to categorize medication stimuli with positive/negative stimuli. So the strength of an association (i.e., whether patients associate taking medication with being sick or becoming healthy) depends on the length of time it takes them to press “A” or “L.” If a patient takes two seconds to categorize medication with the word “positive” and it takes him or her one second to categorize the medication with the word “negative” then one could say that the patient has a stronger negative association with the medication. Patients’ scores on two SC-IATs (positive-negative attitudes and health-sickness associations) were calculated by subtracting the log-transformed response times on the positive categorization block (e.g., medicine-positive or medicine-health) from those of the negative categorization block (e.g., medicine-negative or medicine-sickness). This resulted in difference scores, indicating the relative difference in categorization speed on the different blocks. Scores greater than zero indicate that the patients were relatively faster on positive (health-related) than negative (sickness-related) categorization blocks, which is an indication that their implicit associations are positive (health-related). Scores below zero reflect a reversed response time pattern which indicates relatively negative (sickness-related) associations (see **Box 1**).

**Main procedure of the two Single Category Implicit-Association Tests (SC-IAT).**

Participants were instructed to categorize positive stimuli by pressing the L key of the keyboard with their right hand and negative stimuli by pressing the A key of the keyboard with their left hand. Items included words (*N* = 10) and pictures (*N* = 10). Positive words included, for example, ‘nice’ and ‘happy’ and negative words included ‘stupid’ and ‘sad.’ Pictures included positive illustrations such as a laughing baby or thumbs up and negative illustrations included a crying baby or an illustration of thumbs down. After completing this practice block of trials, the patients were asked to categorize medication stimuli in addition to the previous stimuli. Medication stimuli consisted of pictures of people taking pills, pill boxes and the actual MTX pillbox. Blocks occurred in counterbalanced order, with half of the patients first categorizing medicine items on the same key as positive items (and next making a reversed medicine-negative categorization), and the other half first categorizing medicine items on the same key as negative items. Next, the patients performed a similar task to measure health versus sickness-related associations. The health-related stimuli included words (*N* = 10) and pictures (*N* = 10). The stimuli included health-related words such as ‘vitality’ and ‘energetic’ and sickness-related words such as ‘drained’ and ‘tired.’ Health-related pictures included a fit woman and a little girl eating healthy snacks, and the sickness-related pictures consisted of a sick woman and a little girl with a thermometer in her mouth.

Patients’ explicit medication attitude toward MTX was measured using an 8 items 5-point bipolar evaluative adjective scale (α = 0.91). The 8 items that were used to measure patients’ attitude toward medication were for example ‘beneficial-harmful,’ ‘positive-negative,’ and ‘pleasant-unpleasant.’ An average score was calculated resulting in a scale ranging from 1 to 5. Patients’ explicit medication associations with either health or sickness were assessed with a 13 items 5-point bipolar evaluative adjective scale (Ajzen, 1991) (α = 0.936). The items that were used to measure patients’ medication associations were ‘healthy-sickness,’ ‘energetic-tired,’ ‘strong-limp’ etc. An average score was calculated resulting in a scale ranging from 1 to 5. Higher scores indicated more positive attitudes and health-related associations.

The beliefs about medicines questionnaire (BMQ-specific) was used to assess patients’ explicit beliefs toward medication (Horne et al., 1999). It measures beliefs about the *necessity* of taking their medication (α = 0.87) and patients’ *concerns* about taking their medication (α = 0.52). The patients were able to indicate their level of agreement on a 5-point Likert scale ranging from ‘strongly disagree’ (1) to ‘strongly agree’ (5). The scores on each scale were summed. A necessity-concerns differential (NCD) was calculated by subtracting the concerns score from the necessity score (Horne and Weinman, 1999; Clifford et al., 2008; Menckeberg et al., 2008).

#### Clinical (Laboratory) Outcomes

Erythrocyte Sedimentation Rate (ESR) and C-Reactive Protein (CRP) values were retrieved from patient’s medical file.

#### Self-Reported Adherence

Adherence was measured with a 19-item validated medication adherence scale (Compliance Questionnaire on Rheumatology, α = 0.87), six items were stated negatively and were therefore recoded to yield a positive score. The continuous CQR score was calculated by multiplying the patients’ responses by weight (de Klerk et al., 2003).

### Data Analysis

Descriptive statistics were used to describe the sample and the attitude scores. For the implicit attitudes, 0 was considered midpoint and 3 as midpoint for the explicit attitudes. For the SC-IATs, response times for incorrect responses were recorded as missing values (3%), as well as latencies faster than 500 ms or slower than 5000 ms. The remaining response latencies were log-transformed to account for their skewed distribution. For sake of interpretation the untransformed latencies will be reported (Greenwald et al., 1998). One-sample *t*-tests were used to determine if the attitude scores differed from the midpoint. Because of the explorative character of this study, Pearson’s correlations were used to relate the patients’ explicit and implicit attitudes, associations, beliefs, adherence, clinical outcomes and demographics. Correlations were considered significant if *p* < 0.05 and marginally significant if *p* < 0.1.

## Results

### Patients’ Characteristics

Fifty-eight consenting patients with RA using oral MTX participated. Data of 6 patients (10.3%) were removed because they were aged below 18 years (*n* = 1), had not finished the questionnaire (*n* = 2) or performed the IAT test incorrectly (*n* = 3). The final sample consisted of 52 patients who were on average 58.3 years old (*SD* = 15.8), were moderately to highly educated (77.4%) and 48% was male. The average duration of RA since diagnosis was 11.8 years (*SD* = 12.7) (see **Table 1**).

**Table 1:** Patient characteristics (*N* = 52).

Characteristics		*N* = 52	%
Gender			
	Male	25	48.1
Age		M *(SD)*	58.3 *(15.8)*
Diagnosed in years		M *(SD)*	11.8 *(12.7)*
Educational level	Low	20	42.6%
	Moderate	11	23.4%
	High	16	54.0%
Co-morbidity^1^	1	18	
	2	12	
	>2	18	
Co-medication^2^	1	1	
	2	7	
	3	1	
	4	10	
	>4	33	


*^1^Presence of one or more additional diseases co-occurring with RA. ^2^Number of co-medication used by the patient in addition to MTX.*

### Patients’ Explicit and Implicit Attitudes and Associations

Explicitly, the patients reported positive attitudes and health-related associations with their medication, as is indicated by attitude scores above the midpoint of 3 on the 5-point scales (*M* = 3.7, *SD* = 0.9; *M* = 3.6, *SD* = 0.8, resp.). Or in other words, patients reported to be positive about their medication and associated taking medication with being healthy. In contrast, implicit attitudes toward medication were generally negative and their associations sickness-related (*M* = -59, *SD* = 286; *M* = -68, *SD* = 141 resp.). This indicated that, when measured implicitly, patients were negative about their medication and associated their medication with being sick. These implicit attitudes scores were significantly different from 0 [*t*(51)–2.12, *p* = 0.039 resp. *t*(51)–4.04, *p* < 0.001]. A zero score on the IAT indicates that the implicit attitude toward medication was neither positive nor negative. In this case, patients were implicitly significantly negative about their medication (with zero as reference point). Patients explicit and implicit attitudes were not correlated (*r* = 0.08, *p* = 0.59), nor were the patients’ explicit and implicit associations (*r* = -0.13, *p* = 0.35). This indicates that these two types of measures tap into different processes (an explicit and implicit process). Explicit attitudes and associations were related (*r* = 0.77, *p* < 0.001), so were implicit attitudes and associations (*r* = 0.35, *p* = 0.01). Thus, patients who associated their medication with being healthy (respectively explicitly or implicitly) held more (respectively explicit or implicit) positive attitudes toward their medication. Notably, more than half of the patients displayed implicit attitudes that were incongruent with their self-reported explicit attitudes (see **Table 2**).

**Table 2:** Number of patients with congruent and incongruent implicit and explicit attitudes.

	Negative implicit attitude	Positive implicit attitude	Implicit sickness associations	Implicit health associations
Negative explicit attitude	*n* = 2	*n* = 3	*n* = 4	*n* = 1
Positive explicit attitude	*n* = 32	*n* = 11	*n* = 28	*n* = 15
Explicit sickness associations	*n* = 3	*n* = 2	*n* = 3	*n* = 2
Explicit health associations	*n* = 31	*n* = 12	*n* = 29	*n* = 14


### Exploratory Findings

Further inspection of the data showed no significant correlations between self-reported adherence and explicit attitudes (*r* = 0.035, *p* = 0.82), implicit attitudes (*r* = 0.059, *p* = 0.70), or implicit associations (*r* = -0.02, *p* = 0.89). This means that there was no correlation between adherence, patient’s explicit attitudes or implicit attitudes. There was a trend effect (*p* = 0.07) with a small to medium effect size (*r* = 0.28) that explicit associations correlated with self-reported adherence. Thus, the more the patients self-reportedly associated their medication with health, the more adherent they reported to be.

Explicit attitudes and associations positively correlated with beliefs about medicines (*r* = 0.35, *p* = 0.01 resp. *r* = 0.33, *p* = 0.02) indicating that the more positive patients explicitly are, the more they report to perceive that their medication outweighed patients’ concerns about medication. Moreover, explicit attitudes and associations were significantly related to age (*r* = 0.41, *p* = 0.003 resp. *r* = 0.39, *p* < 0.001) indicating that older patients were explicitly more positive about their medication. Concerning patients’ clinical outcomes, a positive significant correlation between explicit attitudes and associations and patients’ ESR was found (*r* = 0.41, *p* = 0.013 resp. *r* = 0.35, *p* = 0.03). In other words, patients with a higher inflammatory activity were more positive about their medication. Implicit attitudes correlated marginally with explicit beliefs (*r* = 0.26, *p* = 0.08) indicating that patients with more positive implicit attitudes report to perceive that their medication outweighed patients’ concerns about medication. Lastly, implicit associations were marginally significantly related to the years since diagnosis (*r* = 0.27, *p* = 0.05) indicating the longer the patients were diagnosed, the more their medication was implicitly associated with health (versus sickness; see **Table 3**).

**Table 3:** Correlations.

	Implicit attitudes	Implicit associations	Explicit attitudes	Explicit associations	Beliefs about medication	Adherence	Age	Years since diagnosis	Erythrocyte Sedimentation Rate
Implicit associations	*r* = 0.347^∗1^								
Explicit attitudes	*r* = 0.075	*r* = -0.115							
Explicit associations	*r* = 0.139	*r* = -0.132	*r* = 0.765^∗∗^						
Beliefs about medication	*r* = 0.255^#^	*r* = -0.146	*r* = 0.346^∗^	*r* = 0.328^∗^					
Adherence	*r* = 0.059	*r* = -0.021	*r* = 0.035	*r* = 0.276^#^	*r* = 0.191				
Age	*r* = -0.012	*r* = -0.101	*r* = 0.409^∗^	*r* = 0.389^∗∗^	*r* = 0.327^∗^	*r* = 0.167			
Years since diagnosis	*r* = 0.068	*r* = 0.271^#^	*r* = 0.146	*r* = 0.174	*r* = 0.324^∗^	*r* = 0.228	*r* = 0.357^∗^		
Erythrocyte Sedimentation Rate	*r* = 0.264	*r* = 0.059	*r* = 0.411^∗^	*r* = 0.354^∗^	*r* = 0.235	*r* = 0.196	*r* = 0.219	*r* = 0.254	
C-Reactive Protein value	*r* = -0.029	*r* = -0.003	*r* = -0.177	*r* = -0.075	*r* = -0.006	*r* = 0.186	*r* = -0.297	*r* = 0.055^#^	*r* = 0.633^∗∗^


*^1^Pearson correlation coefficient where 1 is total positive correlation, 0 is no correlation, and -1 is total negative correlation. ^#^Marginally significant at *p* < 0.1. ^∗^Statistically significant at *p* < 0.05. ^∗∗^Statistically significant at *p* < 0.001.*

## Discussion

The aim of this study was to assess RA patients’ explicit and implicit medication attitudes and associations, and to explore their relation to self-reported beliefs, medication adherence, and clinical outcomes. Explicitly, patients reported positive attitudes and health-related associations with their medication. Implicit medication attitudes and associations were, however, generally negative and sickness-related. Patients’ implicit and explicit attitudes did not correlate, which indicates a simultaneous but conflicting activation of two different processes (Nosek, 2005). Based on these results, we argue that only addressing explicit attitudes when improving medication adherence might overlook patients who have conflicting attitudes; patients who are explicitly positive but implicitly negative regarding their medication, which constitutes no less than *50 percent* of the current patient sample. Patients who show this pattern of incongruent responses are either unaware of their implicit negativity or are subject to response biases, and are therefore unable to report it explicitly (Dovidio and Fazio, 1992). In a follow-up study with a greater number of patients (higher power) it would be interesting to structurally compare groups (i.e., congruent and incongruent explicit and implicit attitudes) with respect to their behavior and clinical outcomes. With this information, interventions can be developed that target patients’ congruent or incongruent medication attitudes.

Exploratory analyses revealed that in line with previous research (Rüsch et al., 2009), patients’ explicit, but not implicit, associations toward medication are related to self-reported medication adherence. When patients are motivated to deliberately think about their medication intake, which is the case when filling out a questionnaire about medication adherence, deliberate processes such as explicit attitudes take over (Gibson, 2008). Consequently, explicit attitudes assessed via self-report correlated more strongly with self-reported behavior. However, individuals often do not know what influences their behavior. Implicit attitudes have been shown to correlate more strongly with *actual* (versus self-reported) behavior (Dovidio et al., 2002; Galdi et al., 2008; Gawronski and Payne, 2010). In light of the unique effects of implicit attitudes’ on objectively measured (versus self-reported) behavior (Dovidio et al., 2002; Galdi et al., 2008) the results of the present study – predominantly negative implicit attitudes toward medication – might explain the low actual adherence rates found in previous studies (Hetland et al., 2010; Van den Bemt et al., 2012). That is, even though patients often report to be positive about their medication, they might not fully adhere to their treatment, because they inadvertently have negative and sick-related associations with the medication and behave accordingly without being conscious about it. It might be worthwhile to explore the relation between implicit attitudes and adherence which can be measured more objectively with for example Electronic Monitoring devices (Van den Bemt et al., 2012).

Patients with a lower inflammatory activity were explicitly more negative about their medication. Older patients were explicitly more positive about their medication. Our results also show a positive relationship between implicit attitudes and disease duration, indicating that the longer the patients were diagnosed, the more they implicitly associated their medication with being healthy. This result can be explained by the Patient Health Engagement Model (PHE). According to this model, patients may be differently engaged in their health. These differences can be explained by their emotional, cognitive or behavioral mindset. For example, when patients are newly diagnosed, they are not able to fully participate in their health care because of their concerns about their health. However, the longer patients are diagnosed, the more knowledge they gain, and the more emotionally stabilized they are. Consequently, they are more confident that they can manage their disease and might implicitly relate their medication with being healthy (Graffigna et al., 2015). Furthermore, both patients’ explicit and implicit attitudes were positively correlated with patients’ explicit beliefs about medication. It might be that both implicit and explicit attitudes seem to explain some part of patients’ self-reported beliefs and should be further explored in new research.

A limitation of this study is the small sample size and future research should replicate our findings with a larger sample. Another limitation of this study is that we did not use an objective measure for actual (rather than self-reported) adherence. As previous research suggests that measures designed to capture implicit attitudes might predict actual adherence behavior more accurately than explicit self-reported behavior (Blair et al., 2011), future research should explore the predictive value of implicit attitudes for patients’ actual medication intake behavior. With more information about the predictive value of implicit determinants of actual behavior, interventions that improve patients’ implicit attitudes might lead to more functional (adherent) behavior.

The aim of our study was to assess both explicit and implicit attitudes and associations toward medication and we did not explore which factors these implicit attitudes shape. A prominent view of the origins of implicit attitudes holds that they are often a result of repeated/past experiences and developed through socialization processes (Crano and Prislin, 2011). Empirical evidence supporting this view comes from research in other settings than medication adherence. Further research is needed to explore what shapes patients’ implicit attitudes toward their medication.

Up to now, when understanding and predicting medication (non-)adherence, researchers have mainly relied on theories such as the Health Belief Model (Horne and Weinman, 1998), The Common Sense Model of Self-Regulation (Leventhal et al., 2003) and the Theory of Planned Behavior (Ajzen, 1991). These theories are based on the assumption that people must be motivated to take their medication and that patients are active, self-regulating problem solvers. However, these theories only partly explain non-adherence. For example, recent meta-analyses on the predictive value of the Theory of Planned Behavior and the Common Sense Model of Self-Regulation show that these models have little predictive value in explaining medication adherence (Brandes and Mullan, 2014; Rich et al., 2015). Moreover, these theories have been criticized for their focus on rational reasoning (i.e., planned behavior) and the exclusion of associative (unconscious) processes (Sniehotta et al., 2014). By including patients’ implicit attitudes and associations with their medication in addition to the predominantly tested explicit attitudes and associations, we aimed to pinpoint potentially relevant and influential implicit determinants of the patients’ adherence behavior to understand the reported discrepancy between reported attitudes and actual adherence. We have obtained evidence suggesting that the measurement of implicit attitudes and associations provides different information than explicit, self-reported attitudes toward medication. Giving the above, we can conclude that the relationship between implicit attitudes and medication adherence is worth being further explored which may lead to more effective interventions targeted at implicit or incongruent implicit versus explicit attitudes.

## Author Contributions

Each author has been sufficiently involved in this submission to take public responsibility for the work, meaning that each author has made substantial contributions to the conception and design of the study (AL, LV, AW, LD, MV, and BvdB), or acquisition of data (AL, BvdB), or analysis and interpretation of the data (AL, LvdB, AW), drafting the article and revising it critically for important intellectual content (AL, LV, AW, BvdB, LD, MV).

## Conflict of Interest Statement

The authors declare that the research was conducted in the absence of any commercial or financial relationships that could be construed as a potential conflict of interest.
